# Public attitudes toward dementia risk prediction: A mixed‐methods study

**DOI:** 10.1002/alz.14615

**Published:** 2025-05-12

**Authors:** Jacqueline J. Claus, Mathijs T. Rosbergen, Marije J. Splinter, Jolande van Heemst, M. Arfan Ikram, Meike W. Vernooij, Frank J. Wolters

**Affiliations:** ^1^ Department of Epidemiology Erasmus MC University Medical Center Rotterdam the Netherlands; ^2^ Department of Radiology & Nuclear Medicine and Alzheimer Center Erasmus MC Erasmus MC University Medical Center Rotterdam the Netherlands

**Keywords:** dementia, implementation, mixed‐methods, public perspectives, risk prediction

## Abstract

**INTRODUCTION:**

Numerous dementia risk prediction models have been developed, but uptake in clinical practice is poor.

**METHODS:**

We determined public attitude toward dementia risk prediction through the means of a focus group (*n* = 9) and subsequent online survey (*n* = 687). Thematic content analysis was used for focus group data and descriptive statistics for survey responses.

**RESULTS:**

Focus group participants expressed reluctance in knowing dementia risk due to fear, emotional burden, and belief that prevention was impossible. Conversely, practical benefits and maximizing present quality of life motivated others to know dementia risk. Of survey respondents, 66.1% would want to know their 10‐year risk at present, increasing to 82.3% if preventive medication were available. People perceived their 10‐year risk as rather high, yet lower still than their own personal threshold for considering preventive action.

**DISCUSSION:**

Development and implementation of dementia risk prediction tools require attention for personal actionability and emotional impact of risk communication.

**Highlights:**

- Among citizens with a particular interest in dementia, a large group is keen to learn their personal dementia risk, particularly when effective preventive measures are available.
- In focus group participants, hesitancy to learn about dementia risk was common, driven by fear, emotional burden, and doubts about the effectiveness of preventive interventions.
- The majority of survey participants (72%) believed a healthy lifestyle could reduce dementia risk.
- People perceived their 10‐year risk as rather high, yet lower still than their own personal threshold for considering preventive action.

## 1 BACKGROUND

Accurate prediction of an individual's risk of dementia enables personalized approaches to diagnostics, care planning, treatment options, and participation in preventive trials. Over the past 10 years alone, more than 50 new dementia risk prediction models have been developed to identify individuals at high risk, with most demonstrating moderate to high accuracy (area under the curve [AUC] > 0.70).^1^ However, only a small minority of these models have undergone external validation.^1^, ^2^, ^3^ The fraction of prediction models that make it into clinical practice remains very limited, and no tools are routinely used in clinical practice.

The successful adoption of prediction models in clinical settings typically relies on factors related to the prediction tool as well as the health care setting in which it is applied. First and foremost, prediction tools need to align with the needs of patients and health care providers in routine clinical practice. Other relevant factors for uptake are the model's predictive accuracy, the accessibility of obtaining or measuring the predictors in clinical practice, implementation costs, ease and understandability of risk estimates for both patients and caregivers, and actionability on the basis of the results. Given the complexity and challenges involved in communicating dementia risk,^4^ engagement with the target users is essential for the development and implementation of useful risk prediction models in clinical practice. Several studies have examined the attitudes and preferences toward dementia screening, primarily focusing on detecting (prodromal) dementia, rather than predicting dementia risk years before symptom onset.^5^, ^6^ Two qualitative studies previously inventoried the practical and ethical challenges of dementia risk assessment and communication, through task groups and interviews. Observations highlighted the need for reliable information about dementia, along with the psychological considerations of undergoing risk assessment.^7^, ^8^ No published studies have assessed the barriers and facilitators of individualized risk prediction in larger, quantitative study, in which the needs of patients, the advent of commercial tests, and the understandability and actionability of risk information, in particular, require further investigation.^9^

Using a mixed‐methods approach, we aimed to better understand the needs, beliefs, and concerns of community‐dwelling individuals regarding risk prediction of dementia in clinical practice.

## 2 METHODS

### 2.1 Study design, sample, and recruitment

We conducted a mixed‐methods study to explore the perspectives of community‐dwelling individuals on dementia risk prediction models and their implementation in clinical practice. We first organized a focus group with community‐dwelling individuals without dementia (age range 61–74 years), and subsequently sent out a survey.

For the focus group, participants were recruited from the participant panel of the Rotterdam Study, an ongoing population‐based cohort study investigating determinants and occurrence of disease in persons 40 years of age and older. The study started in 1990 and now comprises 17,931 individuals living in the Ommoord suburb of Rotterdam, the Netherlands. The Rotterdam Study has been approved by the Medical Ethics Committee of the Erasmus MC and by the Ministry of Health, Welfare and Sport of the Netherlands, implementing the Population Studies Act: Rotterdam Study. The design of the Rotterdam Study has been described in detail previously.^10^ The participant panel consists of 21 individuals who expressed interest in actively contributing to the study's design, practical aspects, and discussions related to scientific research. For the current focus group, these participants received an invitation letter by email. Of the 21 invitees, 4 did not respond, 7 declined due to lack of interest or time constraints, and 1 participant initially agreed but did not attend. A total of nine participants joined the focus group, and all nine participants provided written informed consent before the focus group commenced.

For the survey, we collaborated with the Dutch Alzheimer's Association patient organization (Alzheimer Nederland; www.alzheimer‐nederland.nl), who distributed the survey through their social media channels, including Facebook, LinkedIn, and X. All respondents to the survey provided online informed consent prior to proceeding to the actual survey questions.

### 2.2 Focus group

A focus group consists of individuals who gather in an informal setting to discuss a specific topic determined by the researcher. Although the facilitators ensure the conversation stays on topic, they take a non‐directive approach, allowing the participants to freely explore various perspectives on the subject.^11^ The focus group was held on July 10, 2024, at the research center of the Rotterdam Study in Ommoord, Rotterdam, the Netherlands. Prior to the focus group, we introduced the topic of dementia, highlighting its occurrence, and our research aim to understand whether people want to know their dementia risk and how they prefer to receive this information. The session lasted 90 min and was facilitated by two medically trained researchers, with two additional staff members from the Rotterdam Study in attendance. One of these staff members actively contributed to the discussion, whereas the other served as the participant coordinator. Before the focus group, we developed a topic list, which was used to structure the session. The list covered four main areas: knowledge about dementia, knowledge about methods for calculating disease risk, attitudes toward dementia risk prediction models, and the implications of knowing one's dementia risk. These topics were introduced using open‐ended questions (see Table SA). The focus group themes were designed based on identified gaps in the literature and prior discussions with health care professionals, including general practitioners, elder care specialists, and geriatricians. The topics were not disclosed to participants prior to the session. The focus group was audio‐recorded, and the recordings were transcribed verbatim independently by two investigators (J.J.C. and M.T.R.). Any coding discrepancies were discussed between the authors for consensus, consulting if needed with a third researcher who was not present during the focus group (M.J.S.). The transcripts were not returned to participants for review or correction.

**RESEARCH IN CONTEXT**

1. **Systematic review**: The authors reviewed the literature using traditional sources (e.g., PubMed), as well as meeting abstracts and presentations, to identify previous studies on the attitudes of the general public regarding dementia risk prediction tools. Relevant references are cited.
2. **Interpretation**: Numerous dementia risk prediction models have been developed, but uptake in clinical practice remains very limited. We found that among citizens with a particular interest in dementia, a large group is keen to learn more about their personal dementia risk, particularly in the presence of effective preventive measures. However, hesitancy remains due to fear, emotional burden, and uncertainty or unfamiliarity about the efficacy of preventive interventions.
3. **Future directions**: These findings highlight the need to involve target users in the development of dementia risk prediction models, to inform their clinical implementation. When using these tools in practice, it is important to address individual preferences, which may range from a simple curiosity about risk information to more practical motivations, such as preparing family and friends, organizing personal affairs, or making lifestyle adjustments.

### 2.3 Survey

To quantitatively assess the perspectives gathered in the focus group within a larger population, we developed a survey consisting of 15 questions, enabling broader validation of the viewpoints discussed in the focus group. After familiarization with the focus group data, investigators J.J.C. and M.T.R. rephrased the focus group results into closed‐ended questions for the survey. We structured the survey into five subsections: (1) demographic information and living situation, (2) perception of personal dementia risk, (3) modifiability of dementia risk through lifestyle, (4) attitudes toward risk prediction, and (5) reasons for wanting or not wanting to know personal dementia risk. We chose a 10‐year cutoff in the survey questions, which was an arbitrary decision, as many studies predict risks within a range of 5–20 years.^1^ We provided a short information text at the beginning of the survey to ensure basic knowledge of dementia and instructions on how to fill out the survey. The questions were subsequently discussed among the research team, after which the survey tested in 10 individuals older than 50 years, from our personal network, resembling the target audience. After minor adjustments, this resulted in the final version of the questionnaire (Supplemental Material C). The survey was distributed as described and remained open for 3 weeks.

### 2.4 Data analysis

Qualitative data analysis was performed according to the guidelines by Braun and Clarke.^12^ Authors J.J.C. and M.T.R. reviewed all codes to identify patterns and generate overarching themes. M.T.R. drafted the initial summary of these themes. J.J.C. then drafted the initial version of the results section, incorporating illustrative quotes from the focus group. This summary was then reviewed against the initial codes by three researchers (J.J.C., M.T.R., and M.J.S.), and revised accordingly to provide the best possible reflection of the focus group discussion. Coding was performed using Atlas.ti version 22.

We used descriptive statistics to summarize the baseline characteristics of the focus group participants and survey respondents, as well as to quantify survey responses. Proportions, along with their 95% confidence intervals (CIs), were computed using the standard normal approximation. Proportions were compared using chi‐square tests, and medians were compared using the Wilcoxon rank sum test. We performed logistic regression analyses to quantify the associations between^1^ wanting to know dementia risk and age,^2^ participants’ belief that “nothing could be done to reduce dementia risk” and their level of knowledge about dementia (categorized as low = 0, average = 1, high = 2), and^3^ participants’ desire to know their dementia risk and their perceived 10‐year dementia risk. All statistical analyses were conducted using R (version 4.3.2), with the significance level set at α = 0.05.

### 2.5 Research team and reflexivity

The focus group discussions were facilitated by J.J.C. and M.T.R., both of whom are PhD candidates specializing in dementia risk prediction. J.J.C. is a White female medical doctor and epidemiologist from the Netherlands, who brings clinical experience to the study with a specific focus on dementia risk prediction. M.T.R. is a White male technical physician and epidemiologist from the Netherlands, who contributes a technical perspective to the study, emphasizing the integration of risk prediction tools into clinical practice. Academic backgrounds of the coauthors include sociology (M.J.S.), epidemiology (M.J.S., F.J.W., M.A.I., and M.W.V.), neurology (F.J.W.), and radiology (M.W.V.).

The backgrounds of the facilitators, including their ongoing PhD work on dementia risk prediction, may have influenced their approach to and interpretation of the discussions. For instance, it may have prompted the researchers to seek reasons that participants would want to know their predicted dementia risk and how they envision this information being used in clinical practice. To move beyond our own perspective, we engaged in discussions with various stakeholders, including medical professionals (e.g., general practitioners, elderly care specialists, and geriatricians) and the patient organization Alzheimer Nederland.

## 3 RESULTS

We included a total of nine participants in the focus group, and 687 people responded to our survey. Focus group participants were older (mean age, SD: 67.2 [3.4] years) than survey respondents (51.1 [13.3] years), and included more men (85.7% vs 55.6%) (Table 1). Most focus group participants and survey respondents were higher educated (Table 1). Focus group participants lived alone (5/9; 55.6%) more often than survey respondents did (112/687; 16.3%).

**TABLE 1:** Demographics.

Characteristic	Focus group participants	Survey respondents
	*N* = 9	*N* = 687
Age (years), mean (SD)	67.2 (3.4)	51.1 (13.3)
Sex, female	5 (55.6)	558 (85.7)
Education		
Primary/lower	0 (0.0)	122 (17.8)
Intermediate	4 (44.4)	218 (31.8)
Higher	4 (44.4)	343 (50.0)
Unknown	1 (11.1)	3 (0.4)
Living situation		
Alone	5 (55.6)	112 (16.3)
With partner	4 (44.4)	499 (72.6)
With children	0 (0.0)	57 (8.3)
Other	0 (0.0)	19 (2.3)

Abbreviation: SD, standard deviation.

From the focus group, we identified three overarching themes, namely (A) motivations for willingness to know dementia risk, (B) reasons for avoiding dementia risk information, and (C) considerations for clinical implementation of risk prediction models (Table SB). In the next paragraphs, we first summarize the survey results on dementia knowledge and risk perception. We then explore the focus group themes in more detail, integrating the qualitative results from the focus group discussions and the quantitative survey findings.

### 3.1 Knowledge of dementia risk reduction

**FIGURE 1:**
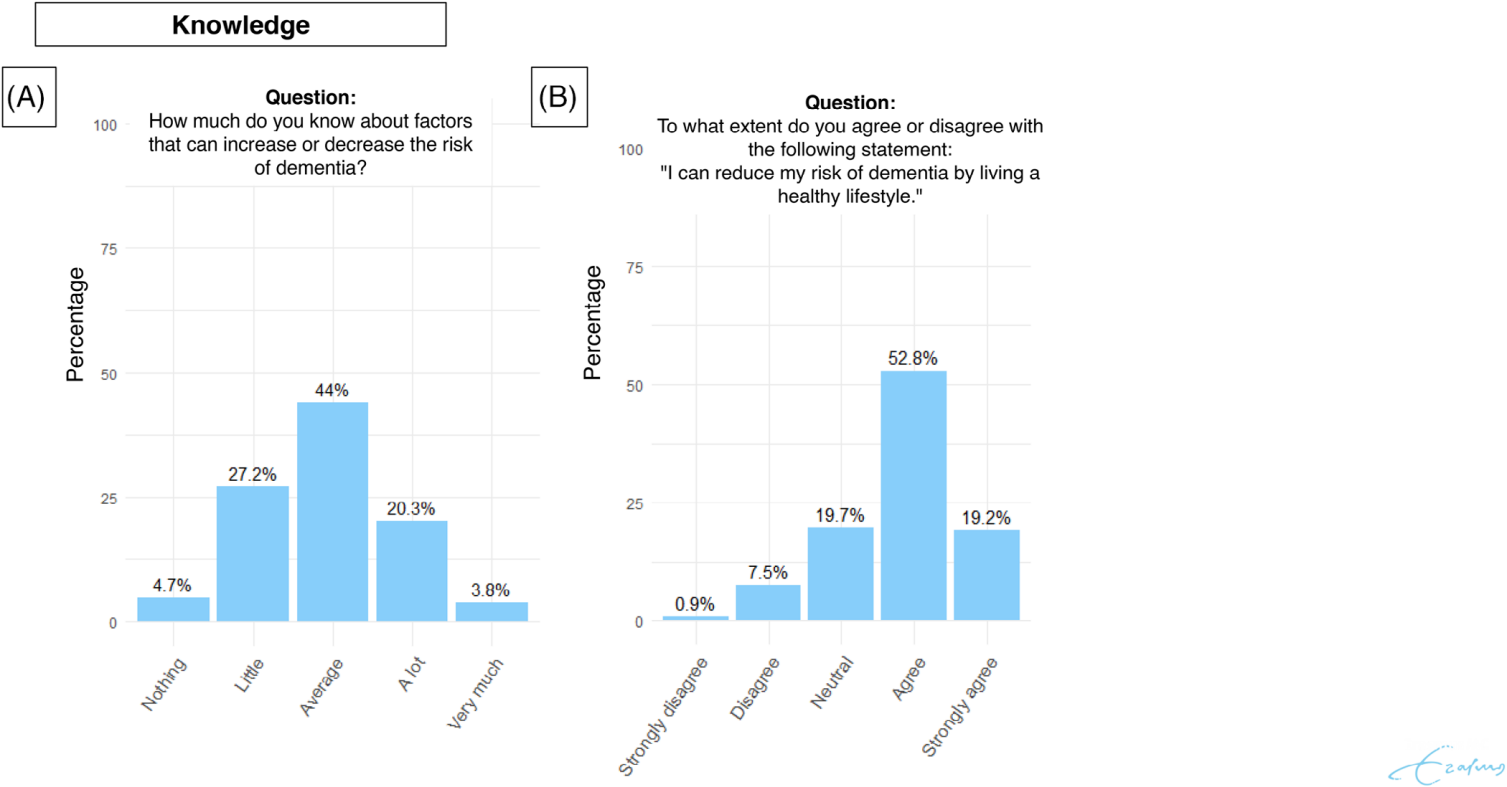
Survey response distribution on questions about knowledge of dementia. Distribution of responses to survey questions among respondents (*N* = 687). Percentages indicate the proportion of respondents selecting each response option.

Of all 682 survey respondents, 299 (43.8%) rated their knowledge of factors influencing dementia risk as average, whereas 181 (26.9%) rated it as lower than average, and 202 (29.6%) as higher than average. The majority of survey respondents (492/684; 72.0%) agreed to the proposition that they could reduce their risk of dementia by living a healthy lifestyle (Figure 1). This pattern was consistent across age and sex, but differed with educational attainment. Respondents with a higher education more often agreed that a healthy lifestyle can reduce dementia risk (287/343; 84.4%) than those with intermediate education (134/218; 61.5%) or primary/lower education (69/122; 56.6).

### 3.2 Dementia risk perception

**FIGURE 2:**
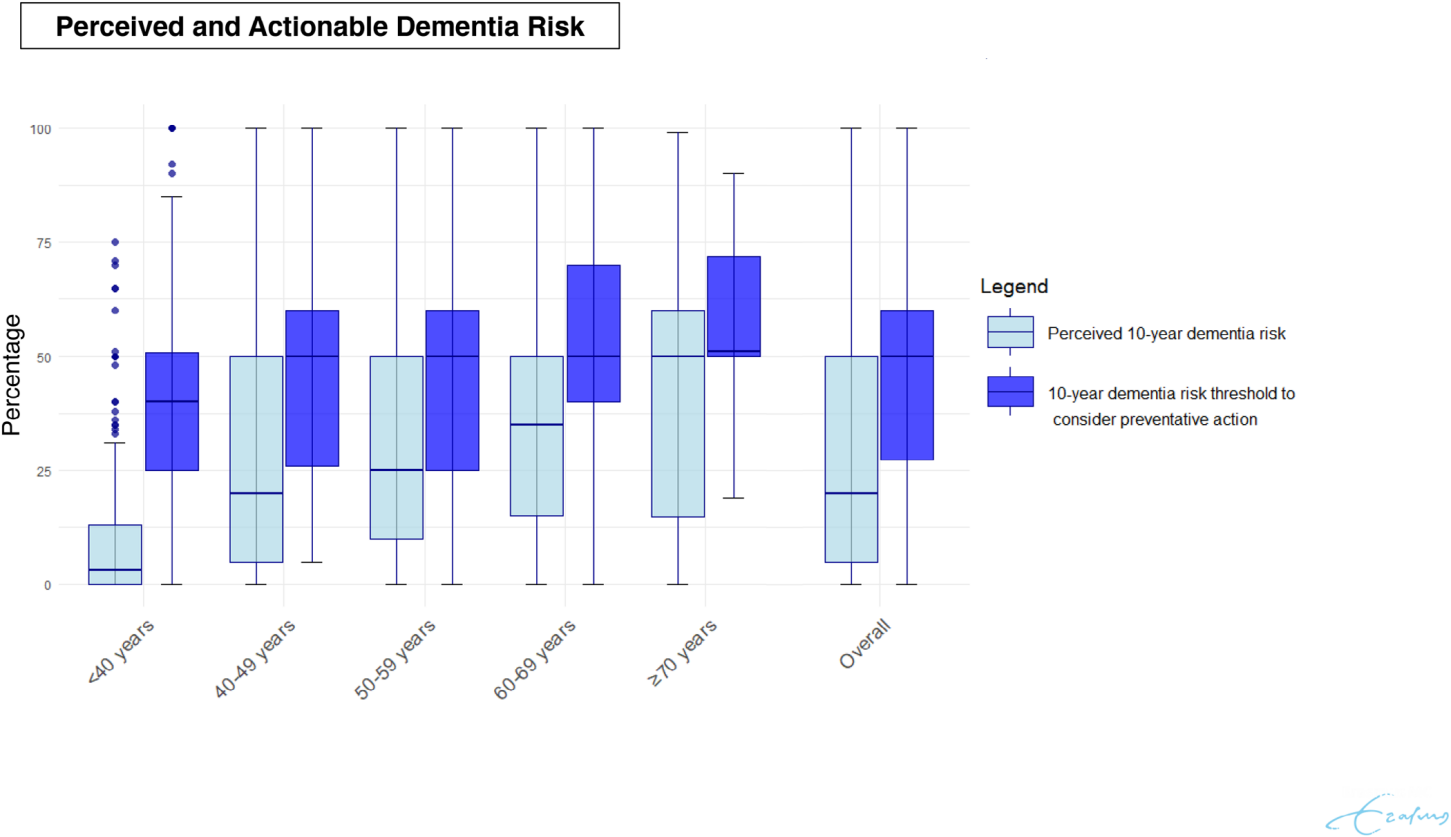
Perceived 10‐year risk of dementia compared to personal thresholds at which one considers preventive measures, among survey respondents. Boxplots represent the distribution of responses to survey questions (*N* = 453), stratified by age. The number of respondents per age category are: <40 years (*N* = 147), 40–49 years (*N* = 132), 50–59 years (*N* = 231), 60–69 years (*N* = 121), >70 years (*N* = 56), and the overall population (*N* = 453). Whiskers extend to the 95th percentiles, with data points beyond this range plotted as individual observations. Perceived risk was inquired by asking: “How high do you estimate your chance of developing dementia in the next 10 years?” Risk thresholds refer to the question: “At what level of dementia risk would you take measures to reduce your risk?”

Survey respondents estimated their 10‐year risk of developing dementia at a median of 20.0% (interquartile range [IQR] 5.0–50.0). Perceived risk increased with age, ranging from 11.4% (IQR 3.0–13.0) in respondents younger than 40, to 50.0% (IQR 14.8–60.0) in individuals of 70 years or older (Figure 2). Most survey respondents (299/682; 43.8%) estimated their dementia risk to be similar to others of the same age, whereas 202 (29.6%) thought their risk was higher (24.5% slightly higher and 5.1% much higher) and 181 (26.6%) considered it lower (15.7% slightly lower and 10.9% much lower). Perceived risk was closely linked to how respondents compared themselves to their peers. Those who believed their risk was much higher than their peers estimated their 10‐year dementia risk at 65.0% (IQR 50.0–77.5), whereas estimates were lower among those who saw their risk as only slightly higher (36.0%, IQR 14.5–56.5), similar to others (17.0%, IQR 5.0–50.0), slightly lower (15.0%, IQR 3.5–35.0), or much lower (2.5%, IQR 0.0–18.5). Education also played a role in risk perception. Respondents with higher education estimated a lower 10‐year dementia risk (12.0%, IQR 2.0–50.0) compared to those with only primary education (40.0%, IQR 11.0–57.0), lower intermediate education (47.5%, IQR 7.25–50.0), or intermediate education (25.0%, IQR 2.0–50.0). Respondents who believed that a healthy lifestyle could reduce dementia risk did not perceive their own risk as very different compared to those who did not believe in the modifiability of dementia risk (26.0%, IQR 22.1–29.9 vs 31.6%, IQR 25.0–38.2, respectively). These patterns did not differ with age or between men and women. Participants with a higher perceived 10‐year dementia risk were significantly more likely to want to know their dementia risk [odds ratio (OR) per 10% increase in perceived dementia risk: 1.14, 95% confidence interval [CI] 1.07–1.22; *p* < 0.001]. Of 687 survey respondents, 570 indicated a “risk threshold” at which they would take measures to reduce their dementia risk. This threshold was median 50.0% (IQR 27.3–60.0%), which in the vast majority of respondents was higher than their perceived 10‐year risk (Figure 2). This was consistent across age groups.

### 3.3 Desire to know dementia risk

**FIGURE 3:**
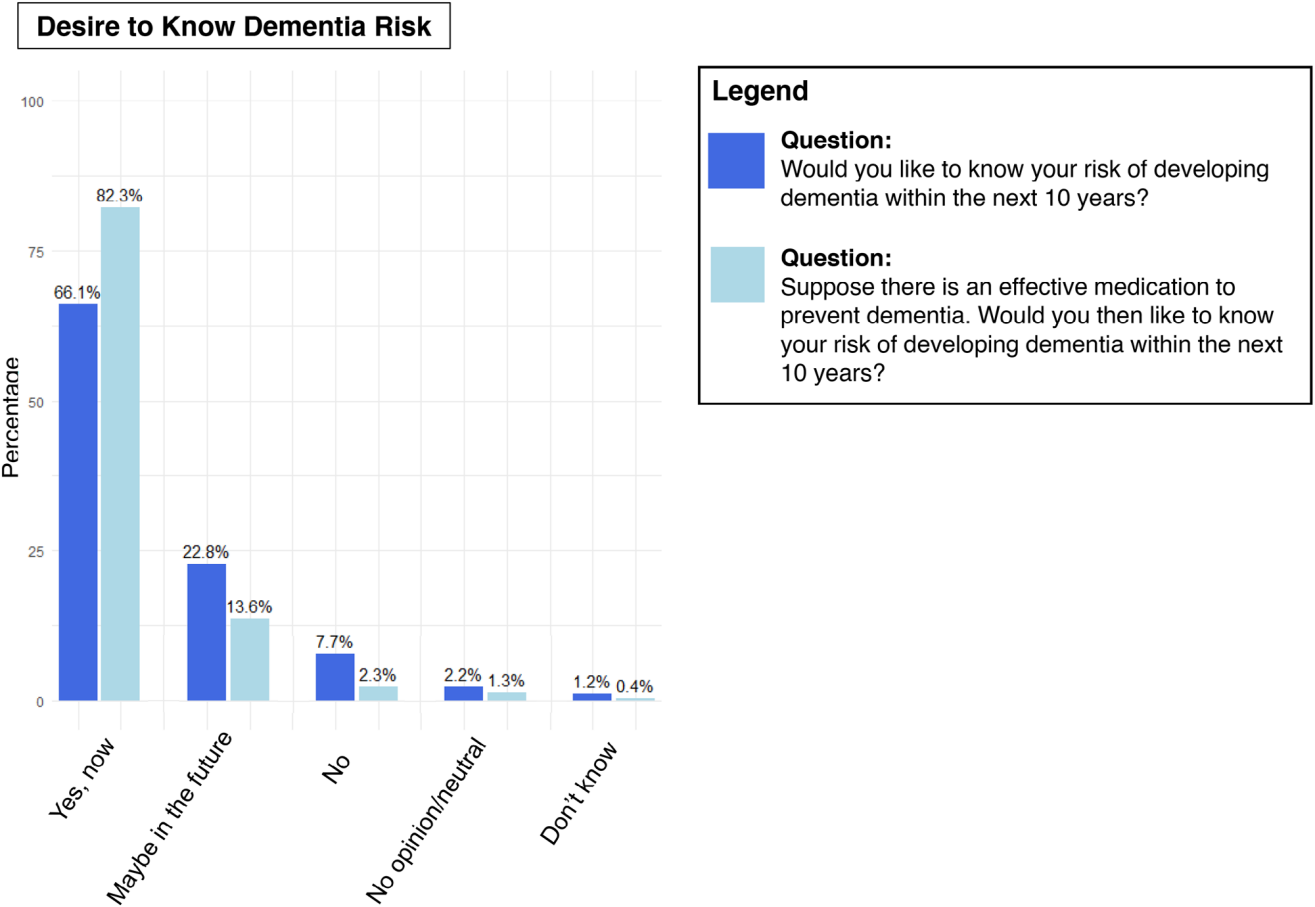
Desire to know one's dementia risk among survey respondents. Distribution of responses to survey questions among respondents (*N* = 687). Percentages indicate the proportion of respondents selecting each response option.

A total of 451 survey respondents (66.0%) expressed a desire to know their dementia risk, and an additional 156 respondents (22.7%) indicated that they might want to know in the future (Figure 3). Only 53 respondents (7.8%) stated they did not want to know their dementia risk. These numbers did not differ significantly for older respondents (age ≥70: 33/47, 70.2%) compared to their younger counterparts (age 50–69: 239/352, 68.5%; <50 years: 174/279, 62.6%; OR per 10‐year increase: 1.08, 95% CI: 0.96–1.21; *p* = 0.21). Presuming an effective medication to prevent dementia were available, the share respondents interested in knowing their dementia risk increased from 66.0% (451/683) to 82.3% (562/683), with only 2.3% preferring not to know their 10‐year dementia risk (Figure 3).

Prior beliefs about dementia risk reduction were unrelated to the attitude toward risk prediction; individuals who believed lifestyle could modify their dementia risk were just as willing to hear about their dementia risk (65.1%) as those who did not (68.2%). Similarly, participants who perceived their risk factor knowledge as higher than average would like to be informed equally (61.2%) as those with lower than average self‐perceived knowledge (67.6%). Respondents who were interested in knowing their current dementia risk estimated their 10‐year risk slightly higher (median 25%, IQR 6.5–50.0%) than those who were not interested in knowing their risk (median 15.0%, IQR 3.0–40.5%; *p* = 0.667). Results were similar between women and men. Desire to know dementia risk was slightly lower among respondents with a higher education (60.5%), compared to intermediate education (73.4%) and lower/primary education (69.7%).

### 3.4 Reasons for wanting to know one's dementia risk

Participants in the focus groups mentioned several reasons for wanting to know their predicted dementia risk. One key reason was the practical utility of this information (Table 2A.; Quote 1). Another reason was to help prepare family and friends. In addition, some participants emphasized the importance of enjoying the present moment (Table 2A.; Quote 2). Other motivations included having a family history of dementia and wanting to adopt a healthier lifestyle while there is still time to make changes. Curiosity about personal health also emerged as a reason (Table 2A.; Quote 3). However, focus group participants were also unsure how they would react if they heard their personal dementia risk (Table 2A.; Quote 4).

**TABLE 2:** Focus group quotes.

A. Focus group participants reasons for wanting to know dementia risk	
Quote 1	“But for practical reasons, because I am single, […] I might still take some actions.”
Quote 2	“There's nothing more to be done about it. Go and enjoy life.”
Quote 3	“I want to know everything about my body”
Quote 4	“Do you want to know or not? I would think yes, but I have no idea how I would react if I heard it.”

B. Focus group participant reasons for avoiding dementia risk information	
Quote 5	“You can't let it go, and everyone says, 'Hey, enjoy life while you still can,' but you're completely gripped by that fear.”
Quote 6	“The term [dementia] immediately carries a stigma.”
Quote 7	“She [a close friend] was the epitome of healthy living, and yet, she still eventually started experiencing forgetfulness.”

C. Considerations regarding clinical implementation of risk prediction	
Quote 8	“It remains, of course, a very personal choice.”
Quote 9	“But with the possibility of a cure, I think many people would be more inclined to take further steps in general.”
Quote 10	“I would find it very risky to do that [a self‐test] myself.”
Quote 11	“Yes, I would definitely consult with my GP on this to get their perspective. It's important to have another opinion alongside your own.”

**FIGURE 4:**
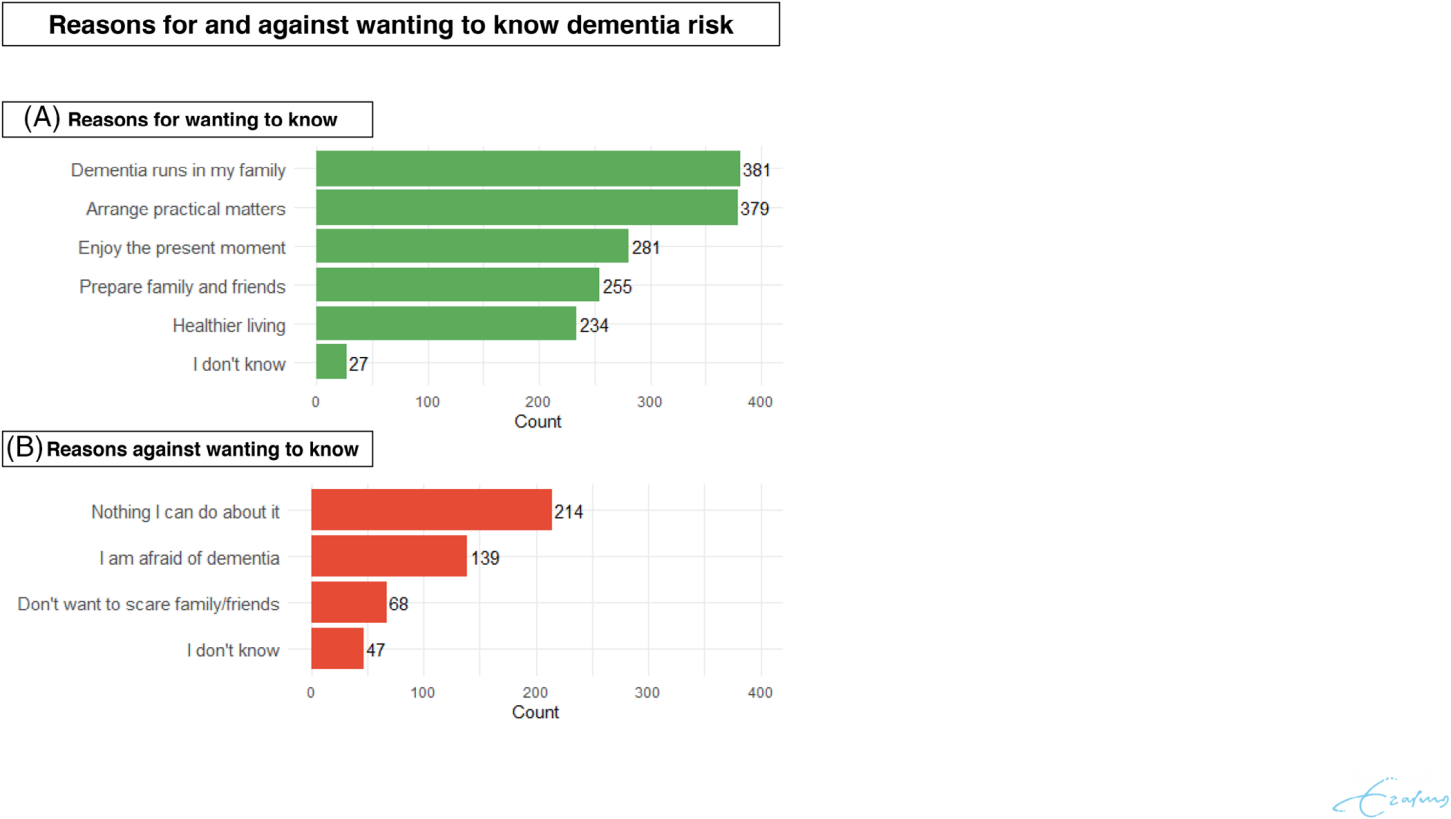
Reasons for and against wanting to know one's dementia risk. Distribution of responses to survey questions among respondents (*N* = 687), for (A) the most important reasons to know dementia risk and (B) the most important reasons against knowing dementia risk. Counts indicate the number of respondents that selected each response option. Individual respondents could provide multiple answers.

In the survey, 381 of 687 respondents (55.5%) indicated “Dementia runs in my family” as a reason for wanting to know their risk (Figure 4). Similarly, 379 (55.2%) stated that knowing their risk would help them manage practical matters, such as preparing a living will or advance directives. In addition, 255/687 respondents (37.1%) wanted to know their risk to prepare family and friends for the possibility of dementia. Finally, 234/687 (34.1%) mentioned that knowing their risk would motivate them to adopt a healthier lifestyle.

### 3.5 Reasons against wanting to know one's dementia risk

Common reasons cited in the focus group for not wanting to know predicted dementia risk included concerns about emotional distress, the fear that an increased risk of dementia could heighten anxiety (Table 2.b.; Quote 5), and the potential for stigma associated with having a high risk of dementia (Table 2.b.; Quote 6). Other reasons against wanting to know dementia risk included the belief that dementia risk cannot be mitigated by lifestyle (Table 2.b.; Quote 7), and difficulties interpreting one's predicted risk. Some participants shared experiences of close relatives who developed dementia despite a healthy lifestyle, reinforcing the belief that preventive actions might be futile.

Around one‐third of survey respondents (214/687, 31.1%) did not want to know their risk because they believed nothing could be done to reduce it (Figure 4). This included 100 participants who earlier agreed to the statement that a healthy lifestyle could reduce dementia risk. Compared to participants with low knowledge, those with average or high knowledge were somewhat less likely to believe that nothing could be done to reduce dementia risk, although the difference was not statistically significant (for average knowledge: OR 0.76, 95% CI 0.52–1.10; and high knowledge: OR 0.81, 95% CI 0.52–1.25). In addition, 139 of 687 respondents (20.2%) reported being afraid of dementia, and 68 of 687 (9.9%) expressed concern about frightening family and friends.

### 3.6 Considerations for clinical implementation of risk prediction

The focus group participants emphasized that deciding whether to know your dementia risk is a deeply personal choice (Table 2.c.; Quote 8), including the timing of when one wants to obtain this information. Most participants indicated a willingness to undergo more invasive testing, if there was a possibility of reducing their dementia risk (Table 2.c.; Quote 9). The idea of a self‐test was put forward, but focus group participants were generally opposed to a self‐test for dementia (Table 2.c.; Quote 10), preferring a more personalized approach to learning about their risk from their health care provider (Table 2.c.; Quote 11). Similarly, two‐thirds (454/685; 66.3%) of survey respondents preferred to learn about their dementia risk from their health care provider, chiefly from a medical specialist (43.6%) or general practitioner (22.6%). Yet nearly one in five survey respondents would consider a self‐test (129/685; 18.8%).

Supposing their predicted risk would turn out higher than expected, a majority of survey respondents would consider changing their lifestyle and habits (567/687; 82.5%) or taking medication (367/687; 53.4%) in order to reduce their dementia risk. The most frequently chosen actions were more regular exercise (67.8%), participating in memory training (61.3%), maintaining a healthier diet (61.1%), and staying socially active (55.3%). Although half of respondents would be willing to take preventative medication with no or limited side effects, only a small minority indicated they would take medication with significant side effects (41/687; 6.0%).

## 4 DISCUSSION

In this mixed‐methods study, we explored public perspectives on dementia risk prediction and identified key reasons for and against wanting to know one's dementia risk. In the focus group, predominant hesitancy to know one's dementia risk was attributed to fear and emotional concerns, and a perceived lack of actionability. Among the survey respondents, the majority wanted to know their risk, especially if effective preventive medication were available.

Response to the survey was overwhelmingly in favor of personalized risk disclosure, somewhat at odds with the impression from our focus group. Survey respondents were likely more interested in dementia due to their engagement with the Dutch Alzheimer's Association, whereas focus group participants were drawn from a population‐representative cohort and were not selected based on dementia interest. Despite the researchers’ intended objectivity during the focus group, their presence or introductory talk may have steered participants in a certain direction. This did not apply to participants in the online survey. The survey respondents represent a subgroup of the population that can be effectively reached with education on dementia risk. Nearly three quarters believed a healthy lifestyle could help reduce dementia risk, which supports the notion that this may be a relatively well‐informed group.^13^, ^14^, ^15^ Yet, survey respondents generally estimated their 10‐year dementia risk much higher than commonly cited 10‐year risks in the general population, with an average estimate of 19.0% among those under 50 and more than 50% after age 70. In reality, 10‐year dementia risk at age 50 is generally <1%, and ranges between 12% and 35% at age 80.^16^, ^17^, ^18^ Accurate risk information could help alleviate public anxiety among those overestimating their own predicted dementia risk. Indeed, respondents who wanted to know their predicted risk, perceived their 10‐year risk as higher than those who did not want to know.^19^ At the same time, one should caution that communicating these risks does not inadvertently reduce motivation for beneficial lifestyle changes.

Actionability emerged as an important argument for knowing one's dementia risk in both the focus group and the survey. This aligns with anecdotal information from an earlier group conversation we had with general practitioners, who were more inclined to use risk prediction tools with clear clinical implications. Actionability could relate to lifestyle changes, medication prescription, or sometimes enrollment in clinical trials. If an effective medication were available, nearly all respondents would want to know their dementia risk. This highlights the importance of developing risk assessment tools and risk communication strategies for clinical practice, in anticipation of targeted disease‐modifying therapies.^20^ It should be noted that many survey respondents perceived their own 10‐year risk as too low to take action, which could explain a lack of personal motivation for lifestyle changes, and may also imply that people with mild cognitive impairment at low predicted risk of disease progression could be reluctant toward active treatment. In any case, the often limited knowledge on modifiability of dementia risk underscores the need for greater public education, for example, through public awareness campaigns.^21^

Our findings emphasize that the desire to know one's dementia risk is a deeply personal decision, with individuals citing various reasons for either wanting or not wanting to know. Focus group participants were generally hesitant toward knowing risk, often due to concerns about anxiety over test results and the stigma associated with dementia. Prior studies on public opinion regarding dementia screening (in prodromal stages), rather than risk prediction, have shown similar views, with anxiety about test results and concerns over stigma being common themes.^5^, ^6^ In our study, participants mentioned practical benefits, emotional relief, and the opportunity to maximize present‐day quality of life as motivating factors for knowing their risk. Similarly, in screening studies, individuals expressed belief in potential benefits, the chance to make lifestyle changes, and family pressure for a diagnosis as key motivators.^5^, ^6^

Clear and understandable communication of disease risk to patients and caregivers can be challenging,^22^ and difficulties in interpreting dementia risk came up as a potential barrier during our focus group discussion. This emphasizes the importance of consulting a health care professional to avoid the potential risks of misinterpreting self‐test results, and may underlie the fact that 80% of survey respondents would not consider a self‐test. We believe effective and empathetic communication by health care providers is important to properly address the complexity of interpreting dementia risk and its actionability.^22^ The substantial variation in patients’ personal preferences advocate for shared decision‐making with patients and their relatives.^23^, ^24^, ^25^, ^26^ Percentages and probabilities can be challenging to understand, and the use of visual aids, such as visual arrays, can help enhance the comprehension of risk probabilities.^27^ Disclosure of genetic risk was not explicitly discussed during our focus group, but should be considered explicitly in the clinical setting, in particular because of the wide variety in social acceptability of genetic testing across cultures.

Although our study offers valuable insights into public perspectives on dementia risk prediction, several limitations should also be considered. First, observer bias may have affected the thematic analysis of focus group data, despite the assessment of transcripts by two independent raters. Second, the survey population consisted predominantly of more highly educated women, potentially limiting generalizability. Third, survey respondents were likely more interested and engaged with dementia than the general public, which may be reflected in more knowledge on dementia and a greater desire to know one's predicted risk. Focus group participants were also involved in research and therefore potentially better informed. Fourth, although we aimed to provide an objective introduction of the topic to the focus group participants, our choice of words and research objectives may inadvertently have influenced participants' perceptions, and consequently impacted the survey results. Fifth, we did not extensively discuss potential impact of risk disclosure on insurance, as we deemed this of minor relevance within universal Dutch health care coverage. Implications may differ between health care settings. Finally, we consulted health care providers, including general practitioners and geriatricians, for the development of the focus group topics, but formally investigated opinions only from the general public. Input from other stakeholders, such as clinicians and policymakers, is also important to guide the development and uptake of dementia risk prediction models.

Among citizens with a particular interest in dementia, many are keen to learn more about their personal dementia risk, particularly in the presence of effective preventive measures. However, hesitancy remains due to fear, emotional burden, and uncertainty or unfamiliarity about the efficacy of preventive interventions. Development and implementation of dementia prediction tools can benefit from a keen eye for individual preferences, and tap into actionable reasons for wanting to know one's dementia risk.

## CONFLICT OF INTEREST STATEMENT

The authors declare no conflicts of interest. Author disclosures are available in the Supporting Information.

## Supporting information



Supporting Information

Supporting Information
