# Supplementary material for: Public attitudes toward dementia risk prediction: A mixed‐methods study
Source: Alzheimers Dement. 2025 May 12;21(5):e14615. doi: 10.1002/alz.14615 (PMC12069024; doi:10.1002/alz.14615)
Supplement: Supplementary file 1 — Supporting Information [file ALZ-21-e14615-s001.docx]

**Supplementary material**

**Supplemental Table A. Topic list focus group**

| **Subject** | **Question** | **Possible follow-up questions** | **Aim of question(s)** |
| --- | --- | --- | --- |
| **Knowledge** | What do you know about dementia? | - What do you think are the most common symptoms of dementia? - How likely do you think it is that you will develop dementia? - How likely do you think it is that a 50-year-old has dementia? - How likely do you think it is that an 80-year-old has dementia? | To gain insight into participants' knowledge, awareness, and personal perspectives on dementia. |
| **Knowledge** | Have you ever heard about ways to calculate the likelihood of getting a certain disease? | - Can you give examples of such methods? - What do you think about these methods? - Do you think these methods are useful or not? | To understand participants' knowledge of prediction models. |
| **Attitude** | There are prediction models for dementia, but they are currently mainly used for scientific purposes and not yet in clinical practice. We are investigating whether they could be useful in general practice or hospitals. What are your thoughts on this? | - Can you imagine a situation where you would want to know your risk of dementia? - Would you want to know this for dementia specifically, or are there other diseases you would be interested in knowing your risk for? - What reasons should these models be used for? - Why would you want to know your risk? - Is it to provide clarity about your health? - Would you want to know this only if you have symptoms, or even if you don't have any yet? - Might your interest change in the future? - What if better treatments become available? - How far in advance would you want to know the prediction? - Would you use this service if it required an out-of-pocket payment? - What kind of information would you be willing to provide for this calculation? - Would you be willing to have a blood test? - Would you undergo an MRI scan? | To gain insight into participants' attitudes toward dementia risk prediction models. |
| **Implications** | Suppose the doctor calculates your risk of developing dementia. What would you do with this information? | - What would you do if you were found to have an increased risk of dementia? - What would you do if your risk was 50%? - What would you expect from the person who tells you your risk? - From whom would you want to hear your risk? - Who should administer the test (GP, specialist, yourself)? - At what level of dementia risk would you feel reassured? - At what level of dementia risk would you start to worry? - What do you need to properly understand this information? - Suppose the prediction model says your dementia risk is 90%; how would you interpret that? - Is that risk irreversible? - Would you change your diet? - Would you quit smoking? - Would you take your medication for blood pressure more diligently? - What would you advise your partner/father/mother? | To understand what participants might do with this information, for the benefit of themselves and society. |

**Supplemental Material B. English translation of original survey**

The survey was drafted in Dutch layman language and clarity of questions and provided information was evaluated through a feedback round with two professionals working at Alzheimer Nederland.

**Dementia Risk Prediction**

Welcome to the survey: Dementia Risk Prediction

1 in 5 people will develop dementia. We are getting better at predicting who will and who won't develop dementia. This can be useful to know, but also difficult to understand or process.

Through this study, we want to better understand whether people want to know their risk of developing dementia and how this risk can best be communicated to them. This questionnaire is not being used to determine your risk of dementia.

The questionnaire consists of 15 questions.

Please note: click on 'Done' when you have finished filling out the questionnaire.

--------------------------

**Question 1: What is your sex?**

- Male
- Female
- Prefer not to say
- Other, namely:

**Question 2: What is your age?**

Open ended.

**Question 3: What is the latest level of education you have completed?**

- No diploma or primary school
- Secondary school: MULO, ULO, LTS, household school, mavo, vmbo
- Secondary school: HBS, havo, vwo
- Vocational education: mbo
- Scientific education: hbo, university
- Prefer not to say
- Other, namely:

**Question 4: What is your living situation?**

(Multiple answers are possible.)

- Alone
- With a partner
- With children
- Other

**Question 5****: How high do you estimate the chance that you will develop dementia within the next 10 years?**

Slider from 0-100.

**Question 6: Compared to the average person of your age, how high do you estimate the chance that you will develop dementia within the next 10 years?**

- Much lower
- Somewhat lower
- About the same
- Somewhat higher
- Much higher

**Question 7: How much do you know about factors that can increase or decrease the risk of dementia?**

- Nothing
- A little
- Average
- A lot
- Very much

**Question 8: To what extent do you agree or disagree with the following statement:**

**"I can reduce my risk of dementia by living a healthy lifestyle."**

- Strongly disagree
- Disagree
- Neutral
- Agree
- Strongly agree

**Question 9: Would you like to know how high your risk is of developing dementia within the next 10 years?**

- Yes, I want to know
- Not now, but maybe in the future
- No, I don't want to know
- No opinion / Neutral
- I don't know

**Question 10: Suppose there is an effective medication to prevent dementia. Would you then want to know how high your risk is of developing dementia within the next 10 years?**

- Yes, I want to know
- Not now, but maybe in the future
- No, I don't want to know
- No opinion / Neutral
- I don't know

**Question 11: What are the most important reasons for you to want to know your risk of dementia?**

(Multiple options are possible.)

- Prepare family and friends for dementia
- Dementia runs in my family
- Healthier living (so I can still do something about it)
- Arrange practical matters (e.g., living will or advance directives)
- Enjoy the here and now more
- I don't know
- I don't want to know
- Other, namely:

**Question 12: What are the most important reasons for you to not want to know your risk of dementia?**

(Multiple options are possible.)

- I am afraid of dementia
- I don't want to scare my family and friends
- There’s nothing I can do about it anyway
- I don't know
- I do want to know
- Other, namely:

**Question 13: How would you like to find out about your risk of dementia?**

**(Even if you don't want to know the risk now, we are interested in your answer.)**

- Through a self-test (e.g., an app or finger prick)
- Through a commercial service (e.g., a total body scan)
- Through the general practitioner
- Through a medical specialist (e.g., neurologist, geriatrician)
- No opinion / Neutral
- I don't know

**Question 14: Suppose your risk of dementia is higher than you expected. What would you do to reduce your risk of dementia?**

(Multiple options are possible.)

- Nothing
- I believe I am already doing enough to reduce my risk
- Eating healthily
- Exercising enough
- Being socially active
- Memory training, puzzles, bridge, etc.
- Taking medication with no or mild side effects (e.g., blood pressure pill, cholesterol pill)
- Taking medication with many side effects
- None of the above

**Question 15: At what level of dementia risk would you take measures (as in the previous question) to reduce your risk of dementia?**

Slider from 0 – 100%.

If you do not wish to take any measures, please do not answer this question

**Supplemental Table C. Themes and categories focus group**

| **Themes** | **Subthemes** |
| --- | --- |
| 1. Motivations for willingness to know dementia risk | Practical benefits of knowing the risk |
|  | Alleviating emotional burden of uncertainty |
|  | Curiosity about personal health |
|  | Desire for actionable information |
| 1. Reasons for avoiding dementia risk information | Emotional distress from knowing the risk |
|  | Confidence in current preventive measures |
|  | Belief that risk cannot be mitigated |
| 1. Considerations for clinical implementation of risk prediction | Willingness to undergo invasive testing if it significantly reduces risk |
|  | Preference to learn about dementia risk from healthcare professionals |
|  | Need for thorough and sensitive communication by healthcare providers |
|  | Personal choice regarding the timing of receiving risk information |
